# Evaluation of osteogenic potential of Cissus quadrangularis on mandibular alveolar ridge distraction

**DOI:** 10.1186/s12903-021-01847-y

**Published:** 2021-10-04

**Authors:** Alaa Abdelqader Altaweel, Abdel Aziz Baiomy Abdullah Baiomy, Hazem Shawky Shoshan, Hisham Abbas, Ahmed Abdel-Shakour Abdel-Hafiz, Abd El-Hamid Gaber, Amr Abdelfatah Zewail, Marwa A. M. Elshiekh

**Affiliations:** 1 Oral and Maxillofacial Surgery Department, Faculty of Dental Medicine for Boys, Al-Azhar University, Al Mokhaym Al Daem St., Nasr City, Cairo, 11751 Egypt; 2 Oral and Maxillofacial Surgery Department, Vision Colleges, Jeddah, Saudi Arabia; 3 Oral and Maxillofacial Surgery Department, Faculty of Dental Medicine for Boys, Al-Azhar University, Assuit, Egypt; 4 Oral and Maxillofacial Surgery Department, Faculty of Dentistry, Cairo University, Cairo, Egypt; 5 Oral and Maxillofacial Radiology Department, Faculty of Dentistry, Cairo University, Cairo, Egypt; 6 Oral and Maxillofacial Radiology Department, Vision Colleges, Jeddah, Saudi Arabia; 7 Oral and Dental Pathology Department, Faculty of Dental Medicine for Boys, Al-Azhar University, Cairo, Egypt; 8 Clinical Pharmacology, Department of Clinical Pharmacology, Faculty of Medicine, Menoufia University, Menoufia, Egypt; 9 Basic Sciences Department, Vision Colleges, Jeddah, Saudi Arabia; 10 Oral and Dental Biology Department, Faculty of Dental Medicine for Girls, Al-Azhar University, Cairo, Egypt

**Keywords:** Alveolar ridge, Cissus, Distraction, Osteogenic, Quadrangularis

## Abstract

**Background:**

This randomized clinical trial was designed to evaluate osteogenic potential of Cissus quadrangularis in alveolar distraction to facilitate implant installation.

**Material and methods:**

Twenty patients with atrophic ridge were treated by alveolar distraction. After completing distractor activation, patients were randomly divided into two equal groups according to administered drug (placebo and Cissus quadrangularis group). After a consolidation period, distractors were removed and implants were inserted. Clinical evaluation was done to assess wound healing, and distractor and implant stability. Histological evaluation was performed at time of implant insertion. Radiographic evaluation was performed to assess bone volume and density after distraction, as well as, density and bone loss around implant.

**Results:**

Radiographic and histological results showed that bone formation and maturation of study group were faster than that of control group. There was a significant increased bone density in distracted area and around implant in study group than control group. A significant bone loss at end of consolidation period, and around implant at end of the study was reported in control group than study group.

**Conclusion:**

Cissus quadrangularis administration during the consolidation period is associated with increased osteogenic potential of distracted bone. The histological and radiographic findings of current study proved that Cissus quadrangularis not only enhances rate of new bone formation, but also bone density to withstand the biomechanical requirements of implant placement in a shorter time.

*Trial registration*

This study was retrospectively registered on www.ClinicalTrial.gov: NCT04669795-17\12\2020.

**Supplementary Information:**

The online version contains supplementary material available at 10.1186/s12903-021-01847-y.

## Background

Atrophic alveolar bone is a special challenge which preclude proper implant installation and affect successful long-term results [1, 2]. Decreased alveolar bone height may result in insufficient bone volume for implant installation [2–5]. In these conditions, various techniques such as onlay bone graft [6–8], guided tissue regeneration [9] and vertical alveolar distraction osteogenesis (VADO) are recommended [10].

Main advantage of VADO is increased bone height through new bone formation below distracted segment. Moreover, simultaneous lengthening of associated soft tissues through histogenesis [11]. Despite its advantages, there are some drawbacks such as fibrous union and non-union of the bone, development of infection, development of psychological problems, with main problem of elongated period for bone regeneration [12].

Several trials focused on acceleration of bone regeneration to decrease treatment period, improve quality of bone, and minimize risk of bone non-union. For these objectives, adjunctive modalities such as growth factors, hormones, and electrophysiological tools were investigated. Some of these techniques have given good results, but others studies showed no benefit [13, 14].

It was reported that different herbal agents could enhance bone healing. Cissus quadrangularis (CQ) is a medicinal plant that has been used in a traditional medicine to heal broken bones and injured ligaments and tendons. Also, it is considered as tonic and analgesic agent. Effect of CQ on acceleration of bone healing and remodeling have been reported to be effective and this is attributed to its capacity to induce metabolism and enhance minerals absorption by osteoblasts [15, 16].

Accordingly, the aim of this randomized blinded clinical study was to assess osteogenic effect of CQ in VADO for improving bone quality to facilitate treatment in patients requiring implant placement in atrophic posterior mandibular area.

## Methods

This prospective study, conformed to consort 2010, was done on patients, attending outpatient clinic of Oral and Maxillofacial Surgery Al-Azhar University, suffered from atrophic posterior mandible and requested to restore missing teeth by implants. The study duration was from March 2017 till May 2020 and ended as planned in the suggested study's protocol. Research methods were illustrated to all patients and they signed an informed consent form before the study. The study was performed according to rules of ethics declared by Helsinki, and ethical committee approval was obtained. This study was retrospectively registered under www.ClinicalTrial.gov (study no. NCT04669795-17\12\2020).

Preoperative clinical examination was done for all patients to assess their medical status. Also, intraoral clinical examination was performed to evaluate oral hygiene and amount of keratinized mucosa at areas planed for implant placement. Biochemical investigations were done including serum calcium, phosphorous, and serum alkaline phosphatase levels. Radiographically, orthopantomogram (OPG), and cone beam computerized tomography (CBCT) were used to evaluate bone volume available for implant placement.

Patients were included in this study if they were free from any disease that may affect the healing process. Also, presented with 2–3 missing teeth at least in posterior area and CBCT showed bone height above inferior alveolar canal (IAC) ranged 8-10mm.While, patients were excluded if they suffered from any disease affect tissue healing, bad oral hygiene or heavy smoking patients.

Patients were classified randomly into two equal groups, using online software (https://www.randomizer.org, **Group I**: included 10 patients who were given placebo capsules. While, **Group II**: included 10 patients who were given CQ drug (Hadjod capsules 250 mg, Himalaya Drug company, India, Makali, Bengalure)

All surgeries were achieved under general anesthesia and complete aseptic condition. Before surgery, all patients received prophylactic antibiotic (amoxicillin and clavulanate potassium1gm vial) and antiedematous (Chymotrypsin ampule).

**Fig. 1:**
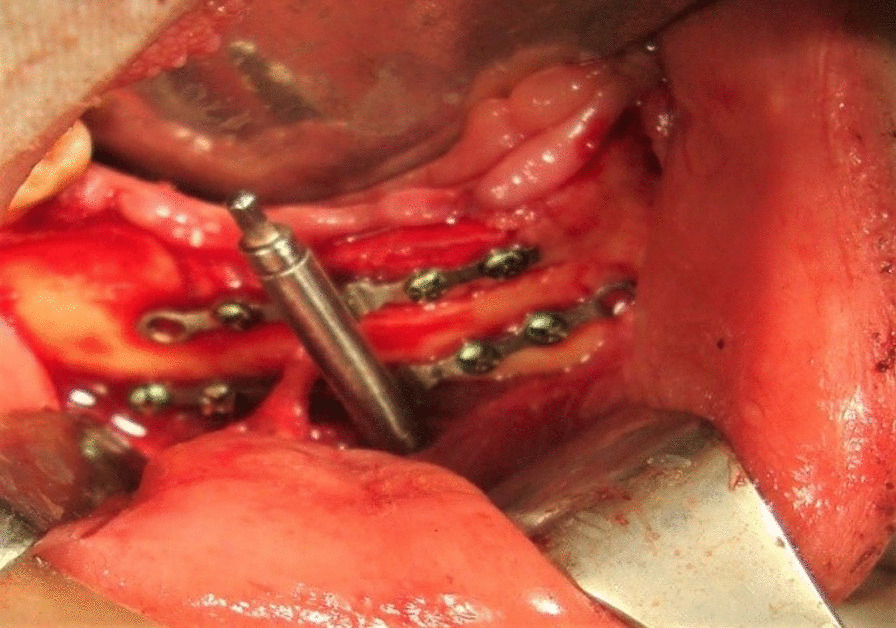
Distractor fixation after osteotomy of transport segment

In all patients extraosseous alveolar distractor (Modern Techniques Centre, Cairo, Egypt) was used. An incision was placed on buccocrestal aspect of alveolar ridge and a flap was elevated. A distractor was placed on the ridge to mark screw sites and then removed. Two vertical bone cuts were made in an angulated manner, and a horizontal osteotomy was done 2 mm above IAC. Bone cut was achieved by bur under copious saline irrigation and completed by osteotome. A distractor was stabilized to basal and osteomatized segments with monocortical 2.0 screws. The basal plate was fixed by 2 screws on either side of distraction rode while the transport plate was fixed with one screw on either side of the distraction rode (Fig. 1). The device was checked for mobility, and wound was closed primarily [17].

The distractor was left for 7 days as latency then it was activated at rate of 1mm/ day (at rhythm 0.5 mm twice), till required height was achieved (overcorrection of 2 to 3 mm was done). A consolidation period extended for 3 months. During the consolidation period, patients were prescribed placebo capsules in control group and CQ capsules in study group. The dose was two capsules once /day with meals for 6 weeks [18].

After the consolidation period, the distractor was removed, and the implants were placed (Super line Systems, Dentium, Co. Ltd, Suwon, South Korea). Implants were loaded 3 months from their installation.

After each surgical stage, clinical evaluation was done to observe signs of infection, wound dehiscence, and function of inferior alveolar nerve. Same preoperative biochemical examinations were repeated immediately after distractor removal.

Histological evaluation was done by biopsy obtained, by a trephine bur, from the implant site during its preparation. The biopsy specimens were fixed in 10% formaldehyde solution in 0.1M phosphate buffer at room temperature for 24 h. Decalcification was done by 20% formic acid for 3–5 days. Specimens were dehydrated in ethanol series (from 70 to 100%) followed by xylene and paraffin tissue processing then embedded in paraffin blocks. Section with 4µ thickness were cut and stained with hematoxylin and eosin for microscopic examination.

Postoperative radiographic evaluation was performed using OPG. Orthopantomograms were taken immediately after distractor placement, at end of the activation period, at the first and second month of consolidation period, and at end of the consolidation period. Change in bone height was evaluated immediately after distractor placement, at end of the activation period, and at end of the consolidation period. Change in bone density was evaluated using Digora software (digora for windows 2.5 Rev 2 copyright © 1997–2007 soredex system from UK system). On every image, mean gray value of marked region of interest (ROI) was calculated as follow:

A Point A was chosen at center of distracted area and pixel density of that point was evaluated on a scale ranged from 0 to 255. A point B was chosen at the same plane, beside the first area, at the sound bone and its pixel density was evaluated.

**Fig. 2:**
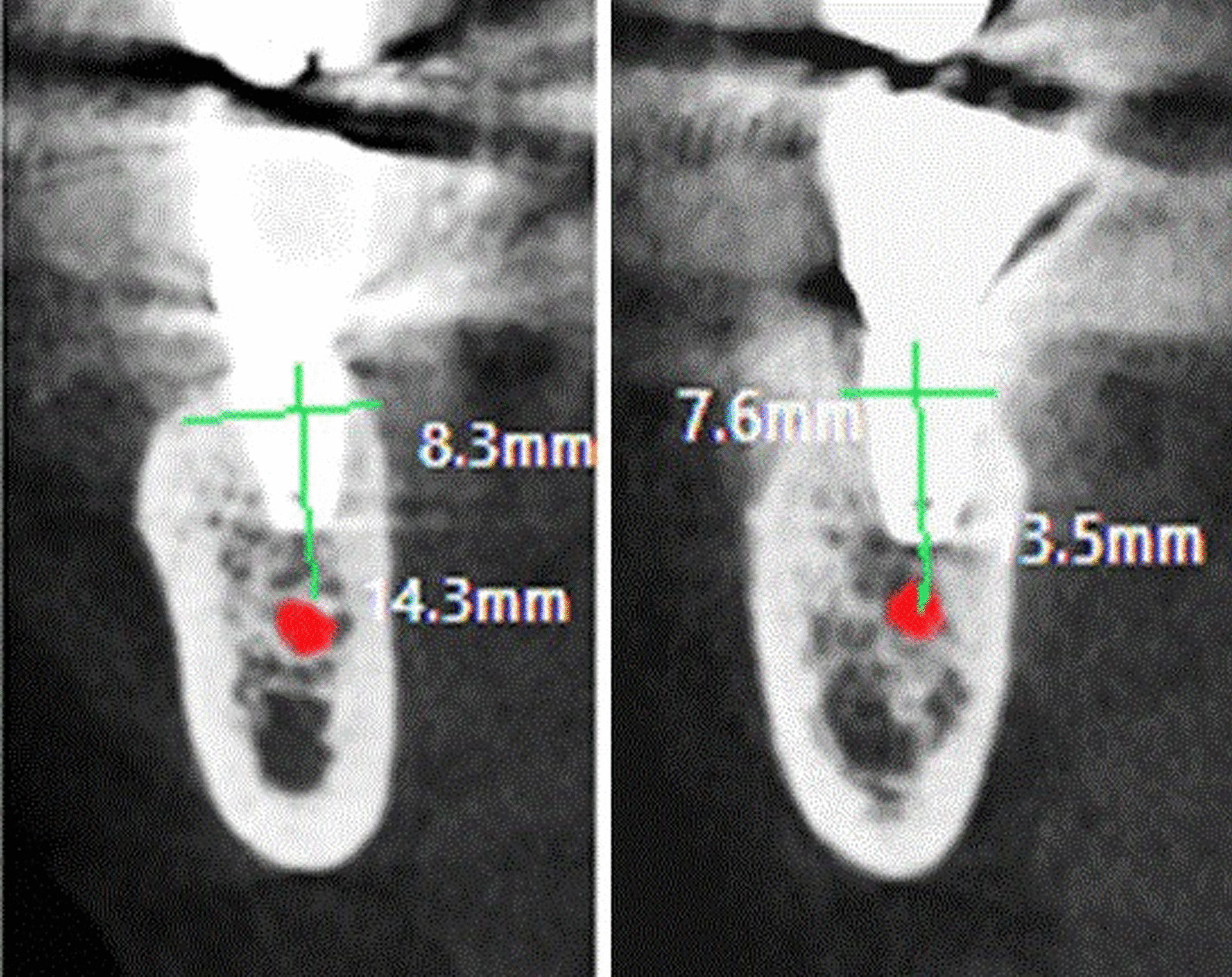
CBCT shows implant after 6 months from its placement

Cone beam computerized tomography was taken immediately after implant installation, and 6 months later (Fig. 2) to evaluate the change in bone density and marginal bone level about the dental implant.

For assessment of crestal bone level around the implant, a distance from implant apex to a point on the implant where bone contacts the implant was evaluated mesially and distally and average of bone loss round each implant was obtained. For bone density assessment, density of bone was evaluated at crestal, middle, and apical portions of the implant using Bioquant® (G Power, Ver. 3.192 copy right 1992–2014) then average was obtained. Region of interest was chosen and traced. Through counting threshold pixels in each ROI, a single pixel that represents a particular color (white pixels in radiographs) was specified permitting automatic selection of all other pixels in ROI that threshold areas are traced and calculated as a number of pixels that can be counted as a ratio of whole ROI.

On multiplanar screen, navigation was done till exact same view position of dental implant was specified on reformatted panorama and cross-sectional cut. Then, crestal bone loss and bone density about implant was assessed.

## Data analysis

Data were recorded and coded on a Microsoft excel sheet, then turned into IBM SPSS statistical software version 21 for demonstrating a descriptive distribution of every variable including frequency, percentage, mean and standard deviation. The paired and unpaired t test were applied to compare continuous variables, significance level was adjusted at ≤ 0.05.

## Results

This study was performed on 20 patients (12 female and 8 male) with mean age of 52 ± 4.1 years. Mean age was 53.6 ± 2.7, and 56.7 ± 1.5 in group I, and group II respectively. Patients of group I were 50% male and 50% female, while in group II they were 60% males and 40% females. There was no statistical difference between both groups regarding age and sex distribution.

### Clinical results

Infection was not reported in this study. Three patients, 2 in group I and one in group II, showed wound dehiscence and plate exposure during distractor activation, and 1 case showed wound dehiscence after second stage surgery in each group. There was insignificant difference between both groups regarding postoperative infection and wound dehiscence.

Lip paresthesia was reported in 9 patients (5 in group I and 4 in group II) after distractor placement which resolved eight to twelve weeks later with vitamin B medication. Regarding lip paresthesia there was insignificant difference between both groups.

**Fig. 3:**
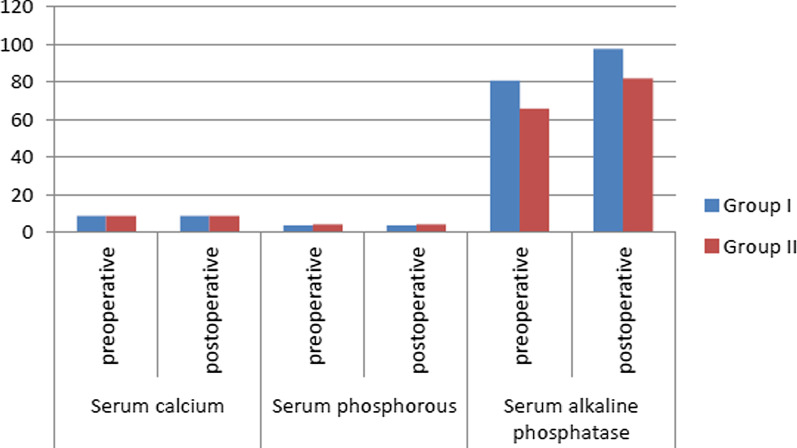
Mean of serum calcium, phosphorous and alkaline phosphatase levels in both groups at different follow up intervals

In both groups, serum calcium and phosphorous levels showed no significant differences along follow up intervals. Alkaline phosphatase level showed a statistically significant rise in group II than group I at all follow-up intervals (*p* = 0.01*) (Fig. 3).

### Radiographic results

In group I, mean bone height was 8.3 ± 2.3 mm before distraction and became 13.6 ± 1.4 mm, and 11.5 ± 1.4 mm at end of activation period and consolidation period respectively. While in group II, it was 8.4 ± 1 mm before distraction and became 13.2 ± 1.5 mm, and 12.7 ± 1.2mm at end of activation period and consolidation period respectively. There was a significant raise in bone height in both groups at end of activation period that followed by significant reduction at end of consolidation period. There was a statistically significant decrease in bone height in group I than group II at end of consolidation period.

**Fig. 4:**
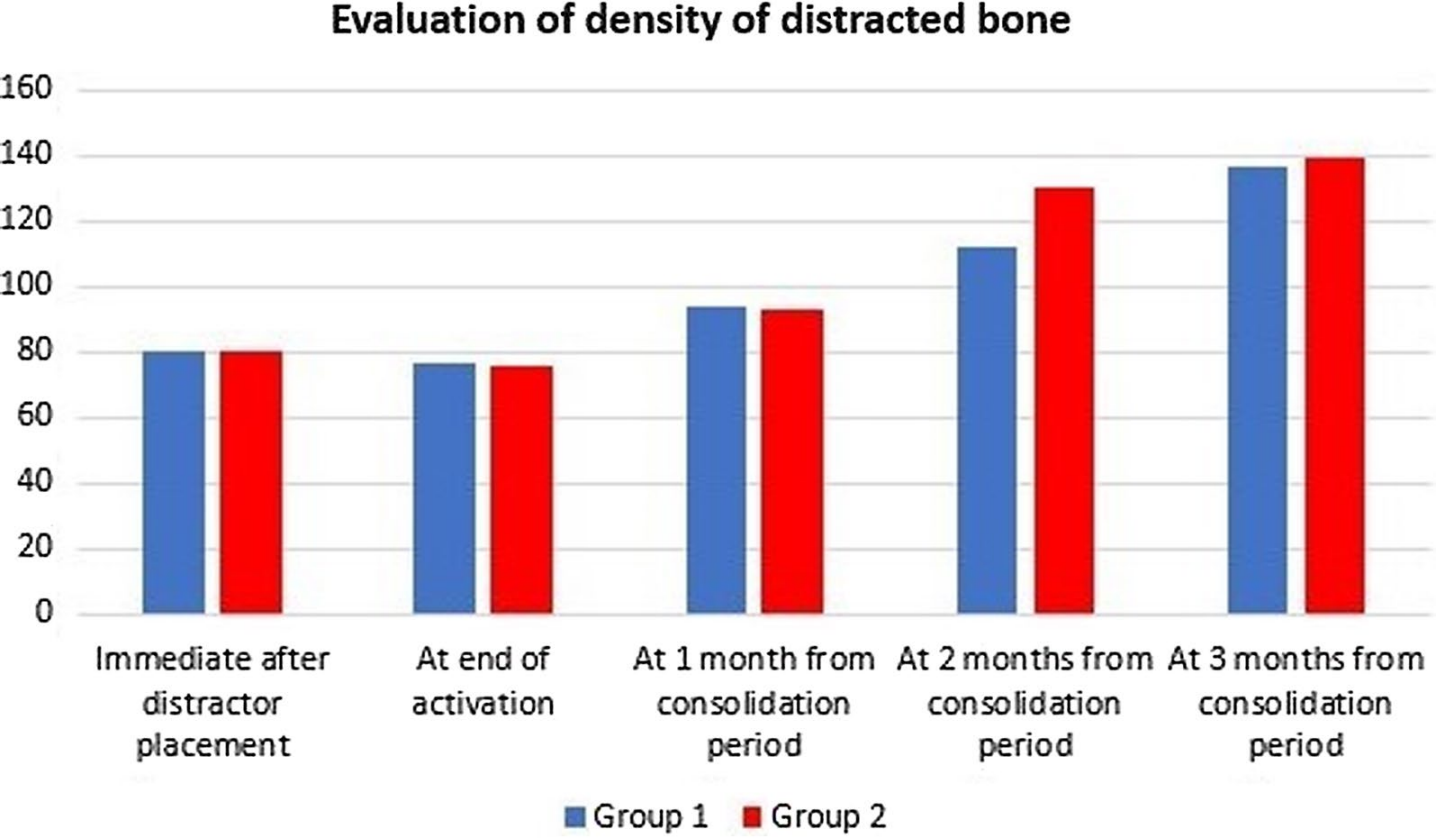
mean of bone density (in HU) at distracted chamber between and inside both groups at different follow up intervals

Regarding bone density of distracted chamber, there was no significant difference immediately after distractor placement, at end of activation period, or at the first month during consolidation period between both groups. At the second month during consolidation period, there was a statistically increased bone density in group II, and insignificant increased density in group I. At end of the consolidation period, both groups showed significant improvement in bone density. Also, there was significant improvement in group II than group I at the second month during consolidation and at end of the consolidation period (Fig. 4).

Marginal bone loss around dental implants immediately after insertion was zero in both groups and increased to 0.94 ± 1.16 mm in group I, and 0.85 ± 1.53 mm in group II after 6 months postoperative. There was insignificant bone loss around implant at end of this study in both groups also, there was insignificant difference between both groups.

In group I, immediately after implant placement mean bone density was 521.80 ± 63.28 Housnfield unit (HU) and increased to 671.31 ± 76.05 HU at 6 months after implantation. While, in group II, mean bone density immediately after implant placement was 582.50 ± 70.54HU and became774.60 ± 138.36 HU at 6 months after implantation. There was a significant increased density in both groups at end of the study. There was a significant increased density in group II than group I immediately implant placement, and highly statistically significant increased bone density (*p* < 0.001) at 6-month interval.

### Histological evaluation

After three months of consolidation period, all specimens showed no signs of forign body reaction or necrosis. Signs of bone formation as presence of active osteoblasts, ostiod material and well vascularized connective tissue were clearly seen.

**Fig. 5:**
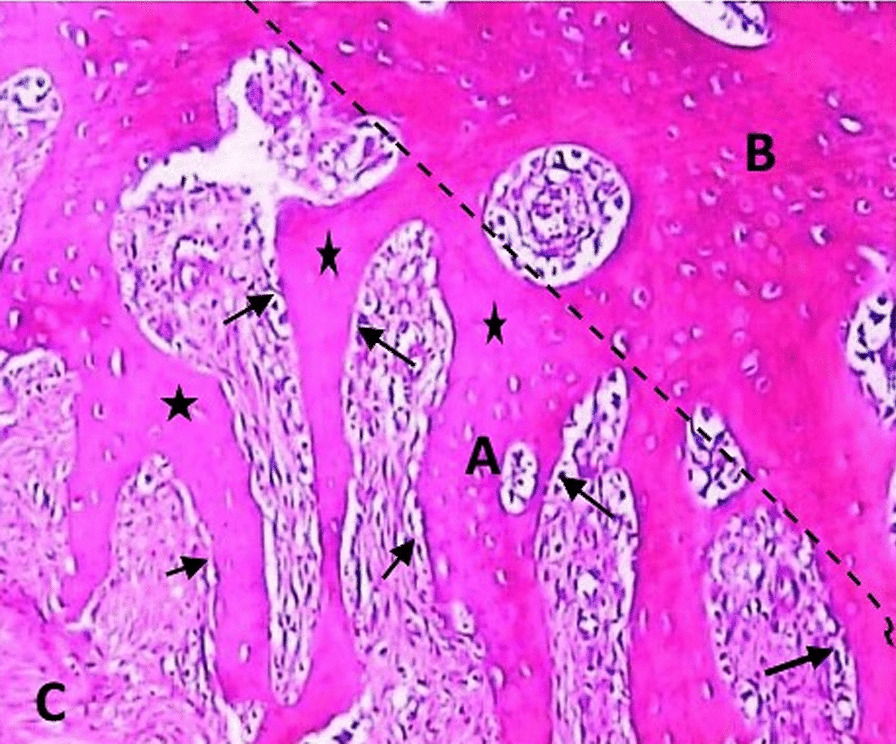
*Group* (I) showing parallel-aligned woven bone (**a**), peripheral bone (**b**), dense fibrous tissue (**c**). Osteoblastic lining (black arrows), large areas of osteoid (asterisk) (H&E orig. mag. × 200)

In group I; distraction gap showed fibrous connective tissue enclosed between longitudinally parallel aligned woven bone trabeculae that were oriented at an angle to surface of peripheral bone. The peripheral bone appeared more maturely arranged than the woven bone which showed few irregulary arranged osteocytes in wide areas of osteoid that were lined by active osteoblastic lining. Deeper towards the centre of the gap dense fibrous tissue was clearly seen (Figure 5).

**Fig. 6:**
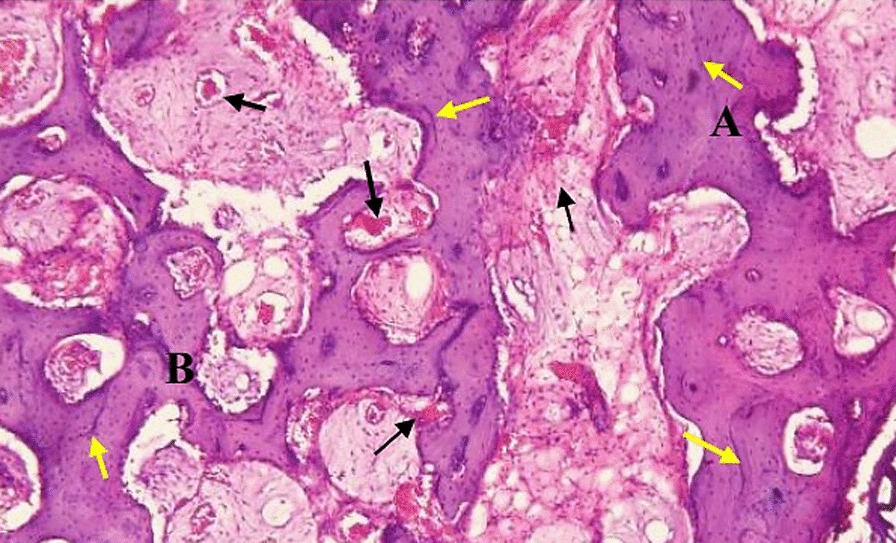
Group (II) interconnected trabeculae; Peripheral (**a**) more mature than central (**b**) marrow spaces (black arrows), resting lines (yellow arrows). (H&E orig. mag. × 200)

In group II; distraction gap was filled with newly formed bone that appeared to have a more mature pattern. The new bone appeared as interconnected thick trabeculae enclosing inside variable sized bone marrow spaces. The peripheral and central bone trabeculae showed no marked interface between them. The medullary cavity occupied by red marrow was clearly observed. The active bone formation was evidenced by the presence of multiple resting lines in the formed trabeculae. The newly formed bone trabeculae showed an organized arrangement of osteocytes giving a lamellar pattern with the peripheral trabeculae showed more mature arrangement than the central ones (Figure 6).

## Discussion

Decreased alveolar bone height is one of the common problems that limits the outcomes of implant rehabilitation and decrease its success rate. Therefore, bone augmentation is mandatory to gain enough bone and good inter-arch relations for superior prosthetic results [2, 4].

Different modalities have been developed to augment atrophic ridge as inferior alveolar nerve lateralization [5], usage of short implants [4, 6] and onlay grafting [7, 8]. However, none of these modalities has achieved satisfactory outcomes to fulfill a successful result.

Distraction osteogenesis allows natural synthesis of bone in comparatively short time. This eliminates the need for graft and decreases treatment time. Soft tissues elongation can accompany bone expansion, this beside the minimal risk of infection as compared to bone grafts [19–21].

Cone beam computerized tomography was used for bone evaluation after implant installation only, and it was not used during different distraction phases to avoid artifacts which may appear due to presence of distractor and also, to limit the use CBCT as its repeated use is harmful. This agrees with Li [22], and Abd-Elaal et al. [23].

This study showed that, a mean increase in bone height was 6.7 ± 1.4 and 6.1 ± 0.9 mm in group I and II respectively. This result is in accordance with Saulacic et al. [24] who reported a mean increase of 6.88 + 2.52mm after VADO, and Pérez-Sayáns et al. [20] who recorded 6.97 to 8.13mm increased bone height.

In this study, an overcorrection of 2–3 mm was carried out to overcome the significant decrease in bone height in both groups throughout all periods that was confirmed by radiographic finding. This bone loss was also reported by Saulacic et al. [24] and Kim et al. [25] who showed 3–30% loss of bone gained after VADO.

There was a significant decreased bone height in group I than group II. Lesser amount of bone relapse in group II may be due to CQ which contains vitamins and steroid, that have the ability to enhance metabolism and increase minerals absorption by osteoblasts, improving bone formation and retarding its resorption. This result matches with Singh et al. [15] and Srivastava et al. [16].

Improved radiopacity of regenerated bone was significant in distracted gap at different intervals during the consolidation period in group II. While a significant improvement was recorded at end of the consolidation in group I. This indicates that newly formed bone in groups II had matured earlier than in group I. This result agrees with Srivastava et al. [16] finding as they observed a decrease in bone healing duration by about 2 weeks after CQ administration. This was proved by our histological findings that showed more bone maturation in group II than group I. This result is in agreement with Deka et al. [26].

Concerning mean crestal bone loss around the implant, it was 0.94 + 1.16 and 0.75 + 1.53 mm in group I and II respectively at 6 months after implantation. This is in agreement with Adell et al. [27] who reported 1.5 mm bone loss around implants during first year. However, there was insignificant difference regarding crestal bone loss between both groups, but group II had smaller value than that of group I. This is in agreement with Bhagat et al. [28] who showed that CQ enhances stem cell proliferation and facilitates osteoblast osteogenesis. Also, this result is in accordance with Murthy et al. [29] report about antioxidant and antimicrobial activities of CQ.

There was a significant improvement in bone density around the implant throughout all follow up intervals with a significant increased density in group II than group I. This result is in accordance with Bhagat et al. [28].

Serum Ca and phosphorus levels were insignificantly decreased in both groups. This is in agreement with Singh et al. [15, 30]. While, alkaline phosphatase level was increased along all periods of study in group II than group I. This is in accordance with Nayak et al. [18]. Increased alkaline phosphatase level is an indicator for increased volume and mineralization of developed callus [18]. Controversially, these findings are in contrast to Deka et al. [26] who reported an early decrease in serum calcium level, and increased alkaline phosphatase level in early time only indicating rapid callus formation in CQ treated group.

This study had some limitations s as small number of samples beside lack of previous studies evaluating CQ as adjunctive modality to accelerate bone healing in VADO. However, our promising findings encourage carrying more experimental and clinical studies to evaluate enhancement action of CQ in maxillofacial distraction osteogenesis is required (Additional files 1, 2, 3).

## Conclusion

Cissus quadrangularis administration during consolidation period is associated with increased osteogenic potential of distracted bone. The histological and radiographic findings of the current study proved that CQ not only enhances rate of new bone formation, but also bone density to withstand the biomechanical requirements of implant placement in a shorter time.

## Supplementary Information

**Additional file 1.** CONSORT checklist.

**Additional file 2.** Study proposal.

**Additional file 3.** Raw Data.

## Data Availability

The datasets used and analyzed in this study are available on reasonable request from the authors (alaarezk77@azhar.edu.eg, abdelazizbaiomy@azhar.edu.eg).

## Abbreviations

VADO: Vertical alveolar distraction osteogenesis
CQ: Cissus quadrangularis
OPG: Orthopantomogram
CBCT: Cone beam computerized tomography
ROI: Region of interest
HU: Hounsfield unit
H&E: Hematoxylin and eosin
