# Supplementary material for: Evaluation of osteogenic potential of Cissus quadrangularis on mandibular alveolar ridge distraction
Source: BMC Oral Health. 2021 Oct 4;21:491. doi: 10.1186/s12903-021-01847-y (PMC8489104; doi:10.1186/s12903-021-01847-y)
Supplement: Supplementary file 2 — Additional file 2. Study proposal. [file 12903_2021_1847_MOESM2_ESM.docx]

**Introduction**

From esthetic and functional points of view, rehabilitation of edentulous patient with dental implant has become the most widely accepted method with long term success rate ^(1)^.

One of the most common problems in oral implantology is insufficient alveolar bone height, which may result from acquired or congenital alveolar bone loss. Acquired alveolar bone loss includes post extraction defect, traumatic avulsion, periodontal disease, tumor ablation, and/or prolonged denture wear with subsequent disuse atrophy ^(1, 2)^.

The edentulous mandible atrophies progressively, losing up to 50%-60% of its original volume, and in severe cases this atrophy affects both the alveolar ridge and the mandibular basal bone. The areas showing most loss are the molar and premolar regions (the posterior mandible) ^(3, 4)^. Insufficient bone height often means that the crown height: implant length ratio is too great, which is likely to reduce the implant’s useful lifespan, because of the leverage effect (degree of force acting on the implant site). It has been suggested that the length of the crown should be no more than 50% of total length of the prosthesis ^(5)^.

Modalities to augment alveolar ridge defects include transpositioning of the mandibular nerve to allow insertion of long stable implants in the molar regions. However, paraesthesia of the mental nerve has been reported ^(6)^. Another technique is to place short implants above the mandibular canal. This technique compromises load-bearing capacity of such implant ^(5, 7)^. Neither of these techniques resolves the problem of excessive length of the crown. Another approach is autologous bone grafting, but such grafts are subjected to resorption, limited to soft tissue tolerance and associated with donor site morbidity ^(8, 9)^.

In recent years, alveolar distraction has gained increasing acceptance to augment alveolar ridge. Alveolar distraction osteogenesis is a surgical technique that encourages creation of new bone and soft tissue through incremental lengthening of osseous segments. The technique is relatively uncomplicated and avoids the need for bone grafting. The secondary effect of stretching and creating new soft tissues, a technique called distraction histogenesis, is particularly helpful in vertical ridge augmentation ^(10, 11)^.

Stable fixation of the osteotomized bone segments is a critical factor in successful distraction. Studies have demonstrated that stable fixation is associated with excellent regenerate bone formation without a cartilaginous intermediate and with complete remodeling after approximately 12 weeks of rigid external fixation. In contrast, decreased device stability has been shown to result in the formation of a cartilaginous intermediate and a significant delay in osseous remodeling ^(12)^.

Therefore, several trials focused on acceleration of bone regeneration to shorten treatment period, improve quality of bone, and minimize risk of bone nonunion. For these objectives, adjunctive modalities as growth factors, hormones, and electrophysiological tools were investigated. Some of these approaches have yielded promising results, but others showed no benefit^(13, 14)^.

Ancient science of medicine describes various herbal preparations that achieve hastening of bone healing. Cissus quadrangularis (CQ) is one of these preparations which used to accelerate healing in fractured bone. Efficacy of CQ on early ossification and remodeling of bones have been reported to be successful due to its ability to stimulate metabolism and increase uptake of minerals by osteoblasts^(15, 16)^.

**Aim of the study**

This study designed to assess osteogenic potential of CQ in VADO for improving bone quality to facilitate treatment in patients requiring implant placement in atrophic posterior mandibular area.

**Material and methods**

This study will be done on patients who are suffering from atrophic posterior mandible and requesting to restore missing teeth by implants. Research methods will be illustrated to all patients and they will be asked to sign an informed consent form before the study. Patients will be selected from those attending outpatient clinics of Oral and Maxillofacial Surgery, Al-Azhar University.

Preoperative clinical examination will be done for all patients to assess their medical condition. Also, intraoral clinical examination will be performed to evaluate oral hygiene (plaque, bleeding, and carries index) and amount of keratinized mucosa at areas planed for implant placement (level of muscle attachment in relation alveolar crest). Biochemical investigations will be done including serum calcium, phosphorous, and serum alkaline phosphatase level.

Radiographically, orthopantomogram (OPG) (Orthophos3, Sirona system), and cone beam computerized tomography (CBCT) (Kodak 9500 cone beam 3D system) will be used to assess bone volume available for implantation.

**Inclusion criteria**

Patients will be included in this study if they are free from any medical condition that may interfere with healing. Also, present with 2-3 missing teeth at least in posterior area and CBCT demonstrates bone height above inferior alveolar canal ranged between 8-10mm.

**Exclusion criteria**

Patients will be excluded from this study if they are suffering from any disease that might affect wound healing, as metabolic or endocrine disorders, poor oral hygiene or heavy smoking patients.

**Patient grouping**

Patients will be classified randomly, using online software (https://www. randomizer.org, into two equal groups according to drug given to patients: Group I: will include 10 patients will be given placebo capsules. While, Group II: will included 10 patients will be given CQ drug (Hadjod capsules 250 mg, Himalaya Drug company, India, Makali, Bengalure)

**Surgical procedure**

All surgeries will be done under general anesthesia and complete aseptic condition. Before surgery, all patients will receive prophylactic antibiotic.

All surgeries will be done under general anesthesia and complete aseptic condition. Before surgery, all patients will receive prophylactic antibiotic (amoxicillin and clavulanate potassium 1gm vial) and antiedematous (Chymotrypsin ampule).

In all patients extraosseous alveolar distractor (Modern Techniques Centre, Cairo, Egypt) will be used. An incision will be placed on buccocrestal aspect of alveolar ridge and a flap will be elevated. A distractor will be placed on the ridge to mark screw sites and then removed. Two vertical bone cuts will be made in an angulated manner, and a horizontal osteotomy will be done 2 mm above IAC. Bone cut will be achieved by bur under copious saline irrigation and completed by osteotome. A distractor will be stabilized to basal and osteomatized segments with monocortical 2.0 screws. The basal plate will be fixed by 2 screws on either side of distraction rode while the transport plate will be fixed with one screw on either side of the distraction rode. The device will be checked for mobility, and then the wound will be closed primarily^(17)^.

The distractor will be left for 7 days as latent period then will be activated at rate of 1mm/ day (at rhythm 0.5 mm twice), till required bone height will be obtained. Consolidation period will be extended for 3 months. During consolidation period, patients will be prescribed placebo capsules in control group and CQ in study group. The dose will be two capsules once /day with meals for 6 weeks according to Nayak et al^(18)^ protocol of drug administration in fracture treatment.

After completing consolidation period, patient will return for distractor removal, and implant installation. Fixed prosthetic restoration for implants will be carried out 3 months after implant placement.

**Clinical evaluation**

After each surgical stage, clinical evaluation will be performed to observe signs of infection (presence of redness, pain, bade odor, and lymphadenitis), wound dehiscence (presence of separated wound edges), and function of inferior alveolar nerve (presence or absence of numbness at area of distribution of IAN). Same preoperative biochemical examinations were repeated immediately after distractor removal.

**Histological evaluation**

Histological evaluation will be done by biopsy obtained, by trephine bur, from implant site during its preparation. The biopsy specimens will be fixed in 10% formaldehyde solution in 0.1M phosphate buffer at room temperature for 24 hours. Decalcification will be done by 20% formic acid for 3-5 days. Specimens will be dehydrated in ethanol series (from 70%-100%) followed by xylene and paraffin tissue processing then embedded in paraffin blocks. Section with 4µ thickness will be cut and stained with hematoxylin and eosin for microscopic examination.

**Radiographic evaluation**

OPGs will be taken immediately after distractor placement, at end of activation period, at 1^st^, 2^nd^ month of consolidation period, and at end of consolidation period. Change in bone height will be evaluated immediately after distractor placement, at end of activation and end of consolidation periods. Also, change in bone density will be evaluated using Digora software. On each digital image, mean gray value of marked region of interest will be calculated using following steps:

Point A will be chosen at center of distracted area and pixel density of that point will be evaluated on a scale from 0 to 255 according to its radiopacity, where most radiopaque is 255, and most radiolucent point is zero scale. A point B will be chosen at the same level and just beside the first area but at sound bone and pixel density of it will be also evaluated as before.

CBCT will be taken immediately after implant installation, and 6 months later to evaluate change bone density and crestal bone level around implant.

For assessment of crestal bone level around implant, a distance from apex of implant to a reference point at implant surface where marginal bone contacts implant will be evaluated mesially and distally and then average of crestal bone loss round each implant will be measured. For assessment of bone density, density of bone will be evaluated at crestal, middle, and apical portions of implant using Bioquant® (G Power, Ver. 3.192 copy right 1992-2014) then average will be obtained. Region of Interest (ROI) will be chosen and traced. Through counting threshold pixels in each ROI, a single pixel that represents a specific color (white pixels in radiographs) will be determined allowing for automatic selection of all other pixels in ROI that threshold areas will be traced and counted as a number of pixels that can be calculated as a ratio of whole ROI.

On multiplanar screen, navigation will be done till exact same-view position of dental implant was determined on reformatted panorama and cross-sectional cut. Then, marginal bone loss and bone density around implant will be assessed.

**Data analysis**

Data will be coded and recorded on a Microsoft excel sheet, then turned into IBM SPSS statistical software version 21 for demonstrating a descriptive distribution of every variable including frequency, percentage, mean and standard deviation. The paired and unpaired t test will be applied to compare continuous variables, significance level will be adjusted at ≤ 0.05.

**References**

1. Garcia-Garcia A, Somoza-Martin M, Gandara-Vila P. et al. Alveolar distraction before insertion of dental implants in the posterior mandible. Br J Oral Maxillofac Surg 2003; 41: 376–9.
2. Laster Z, Rachmiel A, Jensen T. Alveolar width distraction osteogenesis for early implant placement. J Oral Maxillofac Surg 2005; 63: 1724-30.
3. Bras J, Van Ooij CP, Duns JY, et al. Mandibular atrophy and metabolic bone loss: a radiologic analysis of 126 edentulous patients. Int J Oral Surg1983; 12: 309–13.
4. Ulm C, Solar P, LaHood R, et al. Reduction of the compact and cancellous bone substances of the edentulous mandible caused by resorption. Oral Surg Oral Med Oral Pathol 1992; 74:131-6.
5. Rangert BR, Sullivan R. Biomechanical principles preventing prosthetic overload induced by bending. Nobelpharma News 1993; 7: 4-9.
6. Jensen J, Reiche-Fischel O, Sindet-Pedersen S. Nerve transposition and implant placement in the atrophic posterior mandibular alveolar ridge. J Oral Maxillofac Surg 1994; 52: 662–8.
7. Langer B, Sullivan DY. Osseointegration. Its impact on the interrelationship of periodontics and restorative dentistry. Part I Int J Periodontics Restorative Dent 1989; 9: 84–105.
8. Cricchio G, Lundgren S. Donor site morbidity in two different approaches to anterior iliac crest bone harvesting. Clin Implant Dent Relat Res 2003; 5(3):161–9.
9. Clavero J, Lundgren S. Ramus or chin grafts for maxillary sinus inlay and local onlay augmentation: comparison of donor site morbidity and complications. Clin Implant Dent Relat Res 2003; 5(3):154–60.

10. Clarizio L. Vertical alveolar distraction versus bone grafting for implant cases. In: Jensen O, editor. Alveolar distraction osteogenesis, Chicago: Quintessence Publishing; 2002. p. 59–68.

11.Walker DA. Mandibular distraction osteogenesis for endosseous dental implants. J Can Dent Assoc 2005; 71(3):171–5.

12.Aronson J, Harrison, B, Boyd, C M, et al. Mechanical induction of osteogenesis: The importance of pin rigidity. J. Pediatr. Orthop. 1988; 8: 396.

13. Cho BC, Moon JH, Chung HY, Park JW, Kweon IC, Kim IS. The bone regenerative effect of growth hormone on consolidation in mandibular distraction osteogenesis of a dog model. *J Craniofac Surg.* 2003;14(3):417-25.

14. Hamdy RC, Amako M, Beckman L, Kawaguchi M, Rauch F, Lauzier D, et al. Effects of osteogenic protein-1 on distraction osteogenesis in rabbits. *Bone* 2003;33(2):248-55.

15. Singh N SV, Singh RK et al Osteogenic potential of Cissus quadrangularis assessed with osteopontin expression. NatlJ Maxillofac Surg *Natl J Maxillofac Surg.* 2013;4(1):52–6.

16. Srivastava MG S, Nagori BP. Pharmacological and therapeutic activity of cissus quadrangularis: An overview. *Int J Pharm Tech Res CODE (USA)* 2010;4:1298-310.

17. Rachmiel A, Shilo D, Aizenbud D, Emodi O. Vertical Alveolar Distraction Osteogenesis of the Atrophic Posterior Mandible Before Dental Implant Insertion. *J Oral Maxillofac Surg.* 2017;75(6):1164-75.

18. Nayak T, R K. An Assessment of the Osteogenic Potential of Cissus quadrangularis in Mandibular Fractures: A Pilot Study. *J Maxillofac Oral Surg.* 2020;19(1):106-12.
