# Supplementary material for: Evaluation of osteogenic potential of Cissus quadrangularis on mandibular alveolar ridge distraction
Source: BMC Oral Health. 2021 Oct 4;21:491. doi: 10.1186/s12903-021-01847-y (PMC8489104; doi:10.1186/s12903-021-01847-y)
Supplement: Supplementary file 3 — Additional file 3. Raw Data. [file 12903_2021_1847_MOESM3_ESM.docx]

**Raw Data**

**Age and sex distribution in both groups:**

| Serial | **Group I** | | **Group II** | |
| --- | --- | --- | --- | --- |
|  | Age (year) | sex | Age(year) | sex |
| 1 | 56 | F | 55 | M |
| 2 | 51 | M | 57 | M |
| 3 | 53 | M | 58 | F |
| 4 | 54 | F | 56 | M |
| 5 | 53 | M | 57 | F |
| 6 | 51 | F | 58 | M |
| 7 | 55 | F | 56 | F |
| 8 | 56 | M | 57 | F |
| 9 | 54 | F | 56 | M |
| 10 | 53 | M | 57 | M |
| Mean | 53.6 + 2.7 |  | 56.7+1.5 |  |

**Postoperative clinical evaluation**

| Serial |  | **Group I** | | | | **Group II** | | |
| --- | --- | --- | --- | --- | --- | --- | --- | --- |
|  | infection | | Wound dehiscence & plate exposure | Wound dehiscence after 2^nd^ stage | Lip paresthesia | infection | Wound dehiscence & plate exposure | Lip paresthesia |
| 1 | no | | no | no | no | no | no | yes |
| 2 | no | | no | no | no | no | no | no |
| 3 | no | | no | no | no | no | no | no |
| 4 | no | | yes | no | yes | no | no | no |
| 5 | no | | no | no | yes | no | no | yes |
| 6 | no | | no | yes | no | no | no | yes |
| 7 | no | | no | no | yes | no | no | no |
| 8 | no | | yes | no | no | no | no | no |
| 9 | no | | no | no | yes | no | no | yes |
| 10 | no | | no | no | yes | no | no | no |

**Biochemical evaluation:**

**Serum calcium level evaluation:**

| Serial | **Group I** | | **Group II** | |
| --- | --- | --- | --- | --- |
|  | Preoperative | postoperative | Preoperative | postoperative |
| 1 | 8.54 | 8.57 | 8.92 | 9.03 |
| 2 | 8.91 | 8.86 | 8.51 | 8.53 |
| 3 | 9.12 | 9.24 | 8.76 | 8.69 |
| 4 | 8.34 | 8.33 | 9.08 | 9.11 |
| 5 | 9.01 | 8.71 | 8.82 | 8.81 |
| 6 | 8.71 | 8.59 | 8.31 | 8.42 |
| 7 | 8.65 | 8.66 | 9.17 | 9.18 |
| 8 | 8.83 | 8.49 | 9.08 | 8.48 |
| 9 | 8.71 | 8.41 | 8.11 | 8.10 |
| 10 | 8.21 | 8.19 | 8.64 | 8.67 |

**Serum phosphorous:**

| Serial | **Group I** | | **Group II** | |
| --- | --- | --- | --- | --- |
|  | Preoperative | postoperative | Preoperative | postoperative |
| 1 | 4.38 | 4.24 | 3.46 | 3.85 |
| 2 | 2.91 | 3.49 | 4.23 | 4.27 |
| 3 | 4.06 | 4.18 | 3.95 | 4.1 |
| 4 | 3.52 | 3.19 | 4.55 | 4.65 |
| 5 | 4.37 | 3.46 | 4.32 | 3.71 |
| 6 | 2.82 | 2.95 | 3.96 | 3.96 |
| 7 | 2.73 | 2.96 | 3.85 | 3.88 |
| 8 | 3.25 | 3.98 | 3.86 | 4.13 |
| 9 | 3.48 | 3.37 | 3.66 | 3.87 |
| 10 | 3.55 | 3.01 | 4.56 | 3.86 |

**Serum alkaline phosphatase:**

| Serial | **Group I** | | **Group II** | |
| --- | --- | --- | --- | --- |
|  | Preoperative | postoperative | Preoperative | postoperative |
| 1 | 62.74 | 75.64 | 53.32 | 62.04 |
| 2 | 107.13 | 118.51 | 81.92 | 77.91 |
| 3 | 79.72 | 87.9 | 58.91 | 100.07 |
| 4 | 64.21 | 93.82 | 49.41 | 101.02 |
| 5 | 87.33 | 98.92 | 82.01 | 72.5 |
| 6 | 90.61 | 119.00 | 72.91 | 66.85 |
| 7 | 80.19 | 97.33 | 54.91 | 80.26 |
| 8 | 92.15 | 114.25 | 65.21 | 63.91 |
| 9 | 69.78 | 96.36 | 66.71 | 91.21 |
| 10 | 66.91 | 75.01 | 73.21 | 101.32 |

**Radiographic evaluation on OPG after distractor placement:**

**Bone height evaluation on OPG:**

| Serial | **Group I (mm)** | | | **Group II (mm)** | | |
| --- | --- | --- | --- | --- | --- | --- |
|  | Preoperative | At end of activation period | At end consolidation period | Preoperative | At end of activation period | At end consolidation period |
| 1 | 8.13 | 12.72 | 11. 94 | 8.15 | 13.58 | 12.59 |
| 2 | 8.20 | 14.65 | 12.96 | 8.26 | 13.67 | 12.91 |
| 3 | 9.12 | 13.54 | 12.93 | 8.27 | 12.77 | 12.65 |
| 4 | 8.32 | 12.21 | 12.19 | 8.25 | 11.74 | 11.27 |
| 5 | 8.31 | 15.01 | 12.97 | 8.32 | 13.75 | 12.84 |
| 6 | 8.20 | 13.6 | 12.89 | 8.46 | 14.71 | 14.17 |
| 7 | 8.21 | 13.57 | 11.97 | 8.75 | 13.24 | 13.2 |
| 8 | 8.00 | 14.36 | 12.93 | 9.34 | 12.63 | 12.39 |
| 9 | 8.00 | 12.51 | 10.19 | 8.00 | 13.79 | 13.25 |
| 10 | 8.31 | 14.63 | 12.89 | 8.16 | 12.76 | 12.69 |

**Evaluation of density of distracted bone on OPG by Digora**

|  | Group I | | | | | Group II | | | | |
| --- | --- | --- | --- | --- | --- | --- | --- | --- | --- | --- |
|  | Immediate after distractor placement | At end of activation | At 1 month from consolidation period | At 2 months from consolidation period | At 3 months from consolidation period | Immediate after distractor placement | At end of activation | At 1 month from consolidation period | At 2 months from consolidation period | At 3 months from consolidation period |
| 1 | 79.6 | 75.2 | 93.2 | 117.5 | 134.5 | 82.9 | 79.6 | 93.2 | 123.2 | 137.9 |
| 2 | 82.1 | 78.4 | 97.2 | 119.2 | 136.5 | 79.7 | 75.9 | 97.2 | 131.4 | 138.6 |
| 3 | 76.6 | 70.9 | 86.2 | 108.4 | 132.7 | 75.7 | 71.5 | 86.2 | 135.6 | 139 |
| 4 | 82.7 | 80.4 | 94.3 | 116.2 | 139.8 | 85.6 | 82.5 | 94.3 | 132.5 | 139.4 |
| 5 | 80.6 | 75.8 | 95.5 | 120.4 | 132.7 | 77.7 | 72.7 | 95.5 | 129.2 | 138.3 |
| 6 | 76.5 | 72.7 | 86.3 | 105.5 | 137.4 | 81.1 | 77.7 | 86.3 | 122.2 | 134.9 |
| 7 | 83.9 | 79.7 | 99.3 | 119.2 | 135.2 | 76.6 | 72.6 | 99.3 | 126.4 | 143.6 |
| 8 | 80.5 | 77.8 | 94.7 | 108.4 | 139.7 | 78.7 | 73.9 | 94.7 | 135.6 | 139.2 |
| 9 | 75.6 | 72.3 | 91.1 | 106.2 | 138.9 | 81.5 | 77.6 | 91.1 | 137.5 | 140.4 |
| 10 | 85.4 | 81.7 | 99.2 | 99.4 | 136.5 | 82.9 | 78.2 | 99.2 | 131.2 | 142.3 |

**Radiographic evaluation on CBCT after implant placement:**

**Marginal bon loss around dental implant on CBCT**

| Serial | **Group I (mm)** | | **Group II (mm)** | |
| --- | --- | --- | --- | --- |
|  | Immediately after implant placement | 6 months from implant placement | immediately  after implant placement | 6 months from implant placement |
| 1 | 0 | 0.89 | 0 | 0.72 |
| 2 | 0 | 1.73 | 0 | 0.67 |
| 3 | 0 | 2.08 | 0 | 0.69 |
| 4 | 0 | 0.22 | 0 | 0.68 |
| 5 | 0 | 0.27 | 0 | 0.69 |
| 6 | 0 | 0.94 | 0 | 0.67 |
| 7 | 0 | 2.1 | 0 | 0.71 |
| 8 | 0 | 0.24 | 0 | 0.68 |
| 9 | 0 | 0.93 | 0 | 2.38 |
| 10 | 0 | 0.1.73 | 0 | 0.70 |

**Bone density evaluation after implant CBCT**

| Serial | **Group I (HU)** | | **Group II (HU)** | |
| --- | --- | --- | --- | --- |
|  | Immediately after implant placement | 6 months from implant placement | immediately  after implant placement | 6 months from implant placement |
| 1 | 585.08 | 739.44 | 529.74 | 746.32 |
| 2 | 521.79 | 729.11 | 621.93 | 878.54 |
| 3 | 463.53 | 747.36 | 642.17 | 912.96 |
| 4 | 538.71 | 662.66 | 511.96 | 670.24 |
| 5 | 458.52 | 595.26 | 561.74 | 636.24 |
| 6 | 511.93 | 603.98 | 653.04 | 899.12 |
| 7 | 489.61 | 598.33 | 533.9 | 690.78 |
| 8 | 556.42 | 649.01 | 626.43 | 806.75 |
| 9 | 509.61 | 675.37 | 545.64 | 639.7 |
| 10 | 583.02 | 712.12 | 598.33 | 866.21 |
